# Comprehensive Analysis of the Prognosis and Correlations With Immune Infiltration of S100 Protein Family Members in Hepatocellular Carcinoma

**DOI:** 10.3389/fgene.2021.648156

**Published:** 2021-03-18

**Authors:** Susu Zheng, Linxia Liu, Tongchun Xue, Chuyu Jing, Xin Xu, Yanfang Wu, Meixia Wang, Xiaoying Xie, Boheng Zhang

**Affiliations:** ^1^Xiamen Branch, Department of Hepatic Oncology, Zhongshan Hospital, Fudan University, Xiamen, China; ^2^Key Laboratory for Carcinogenesis and Cancer Invasion, Zhongshan Hospital, Shanghai Medical School, The Liver Cancer Institute, The Chinese Ministry of Education, Fudan University, Shanghai, China; ^3^School of Nursing and Health Management, Shanghai University of Medicine and Health Sciences, Shanghai, China; ^4^Center for Evidence-Based Medicine, Shanghai Medical School, Fudan University, Shanghai, China

**Keywords:** S100 protein family, hepatocellular carcinoma, prognosis, immune infiltrates, mutations

## Abstract

S100 protein family members (S100s) are commonly dysregulated in various tumors including hepatocellular carcinoma (HCC). However, the diverse expression, mutation, prognosis and associations with immune infiltration of S100s in HCC have yet to be analyzed. Herein we investigated the roles of S100s in HCC from the Oncomine, Gene Expression Profiling Interactive Analysis (GEPIA), Human Protein Atlas, Kaplan-Meier Plotter, cBioPortal and TIMER databases. Compared with para-cancer tissues, the expression levels of S100A4/S100A6/S100A10/S100A11/S100A13/S100A14/S100P were higher in HCC tissues, while the expression levels of S100A8/S100A9/S100A12 were decreased in tumor tissues. The mRNA levels of S100A2/S100A7/S100A7A/S100A8/S100A9/S100A11 were correlated with advanced tumor stage. Besides, higher mRNA expressions of S100A6/S100A10/S100A11/S100A13/S100A14/S100P were shown to have shorter overall survival (OS), while higher expression of S100A12 was associated with favorable OS. Further, the mutation rate of S100s was investigated, and the high mutation rate (53%) was associated with shorter OS. Additionally, the expressions of S100s were found to be significantly associated with various immune infiltrating cells. Hence, our results showed that S100A6/S100A10/S100A11/S10012/S100A13/S100A14/S100P may be regarded as new prognostic or therapeutic markers and S100s inhibitors may be helpful in the combination of immunotherapies.

## Introduction

Hepatocellular carcinoma (HCC) comprises nearly 80% of primary liver cancer and is the sixth most common and fourth deadly malignancy globally (Bray et al., 2018). Surgical resection or liver transplantation are the only potential measures for radical cure, while these measures are only limited to a small subset of patients. In fact, nearly 75% patients were belonging to middle-late stage of HCC when first diagnosed, and the 5 year survival rate was dismal (Allemani et al., 2015; Katsanos et al., 2017). Thus, novel prognosis or therapeutic biomarkers for HCC are imperative.

S100 protein family members (S100s) are well-known for their calcium-binding properties, and widely involved in a variety of physiological and pathological processes, including inflammation, cell proliferation, apoptosis and cancer development. Until now, there are at least twenty members that have been reported (S100A1-S100A14, S100A7A, S100A16, S100B, S100P, S100G, S100Z) (Allgower et al., 2020). The expression of S100s are commonly dysregulated in human malignancies, including HCC (Bresnick et al., 2015; Xue et al., 2015). The expression and role of S100s display a distinctive pattern in different cancer types. For instance, S100A2 plays a tumor-suppress role in most cancers including breast cancer, melanoma, gastric cancer etc., but may have an opposite role in lung cancer (Hountis et al., 2014). S100s have been shown to have complex roles in HCC. Apart from S100A5, S100A7, S100A7A, S100A12, S100A13, S100A16, S100B, S100P, S100G, S100Z, the rest family members, such as S100A1, S100A2, S100A3, S100A4, S100A6, S100A8, S100A9, S100A10, S100A11, S100A14, and S100P are shown to be expressed in HCC (Wang et al., 2001; Kittaka et al., 2008; Hua et al., 2011; Luo et al., 2013; Yan et al., 2013; Zhao et al., 2013; De Ponti et al., 2015; Tao et al., 2017; Guo et al., 2018). Furthermore, the protein expressions of S100A1, S100A4, S100A6, S100A14 and S100P detected by immunohistochemistry were reported to be associated with short survival of HCC patients. Nevertheless, some S100 family members, such as S100A5, S100A7, S100A7A, S100A13, S100A16, S100B, S100G, and S100Z have been rarely studied in HCC. Moreover, the role of S100s in affecting the immune microenvironment in HCC has yet to be explored. However, due to small sample size in some studies, these results remained questionable. As far as we know, bioinformatics analysis has yet to be operated to discover the distinct properties of S100s in HCC. With the help of integrated bioinformatics analysis, we are now able to analyze thousands of interested genes through multiple databases at one time (Liu et al., 2020). On the basis of in-depth analysis, we analyzed the expressions, mutations, predictive signaling pathways and associations with immune infiltration of different S100s in patients with HCC and further explored their possible functions, different prognostic roles and the associations with immune infiltrates in HCC.

## Materials and Methods

### Ethics Statement

The study was approved by the Ethics Committee of Zhongshan Hospital. All the data were collected from published databases. It was then confirmed that all written informed consent was obtained.

### ONCOMINE Database

Oncomine database, an online database^1^ was utilized to analyze the mRNA expression of 20 S100s in different types of cancer including HCC (Rhodes et al., 2004). Students’ *t*-test was applied to get a *p*-value (cutoff *p*-value: 0.001, cutoff fold change: 1.5).

### GEPIA Dataset

GEPIA, an online tool that contains the RNA expression of 9,736 tumors and 8,587 normal samples from the Cancer Genome Atlas and the Genotype Tissue Expression (GTEx) projects^2^ (Tang et al., 2017). The dataset is capable of providing tumor/normal differential expression analysis, survival analysis, correlation analysis and so on. HCC patients were divided into high and low expression groups according to their median value of mRNA expression. The cutoffs of *p*-value and fold change were defined as following: *p*-value: 0.01, fold change: 1.

### Human Protein Atlas

The Human Protein Atlas, an online tool which contains protein expression data (detected by immunohistochemistry) from 17 different human cancer^3^, was applied to study the protein levels of S100s in HCC (Asplund et al., 2012). Here, we compared the protein expression of different S100s between HCC tissues and normal tissues by immunohistochemistry.

### The Kaplan-Meier Plotter

The Kaplan-Meier Plotter, a website that contains gene expression data and survival information of various cancers^4^ was used to analyze the prognostic value of different S100s in HCC (Gyorffy et al., 2013). The HCC samples were divided into high and low expression groups according to their median value of mRNA expression and then analyzed by Kaplan-Meier survival plot. Statistically significant was considered when a *p* < 0.05.

### cBioPortal

cBioPortal, an online tool that contains multidimentional cancer genomics data^5^ was used to analyze genetic mutations in S100s and their associations with survival (Gao et al., 2013). The HCC dataset which included 360 patients were chosen for mutation analysis. The co-expression and network was also analyzed.

### Gene Ontology (GO) and Kyoto Encyclopedia of Genes and Genomes (KEGG) Analysis

Fifty interactors based on the cBioPortal tool were further explored by GO and KEGG. The functions and predicted pathways were analyzed in the Database for Annotation, Visualization and Integrated Discovery (DAVID)^6^ (Huang et al., 2009). We then used the R package “ggplot”and “stringr” to perform GO and KEGG pathway enrichment analyses.

### TIMER Database

TIMER, an online tool^7^ that contains comprehensive analysis of immune infiltrates in different types cancers was used to analyze the association between S100s and immune infiltrates in HCC. The associations between gene markers and various immune cells were also recorded (Li et al., 2017). Spearman tests were used for analyzing the correlation coefficients.

## Results

### Expression Levels of S100s and Their Relationship With Clinicopathological Paremeters of Patients With HCC

**FIGURE 1:**
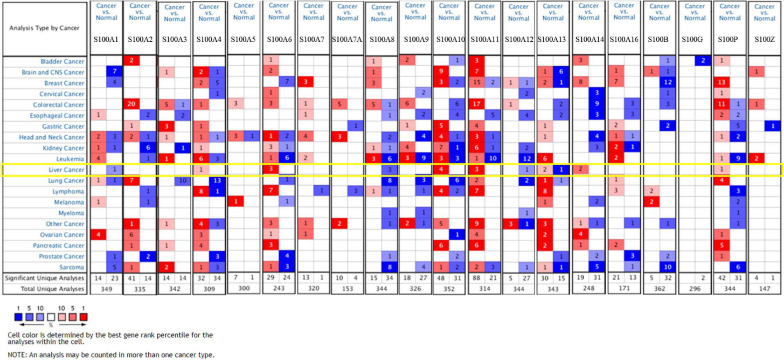
The transcription levels of S100 family members (S100s) in different types of cancers (Oncomine database). Cells in red color represents the overexpression of interested gene, while lowexpression shows in blue. The numbers in these cells represents the numbers of datasets which satisfies the threshold: *p*-value: 0.001, fold change: 1.5. The levels of S100A4/S100A6/S100A10/S100A11/S100A13/S100A14/S100P were higher in tumor than in normal liver samples, while S100A8 were downregulated.

**FIGURE 2:**
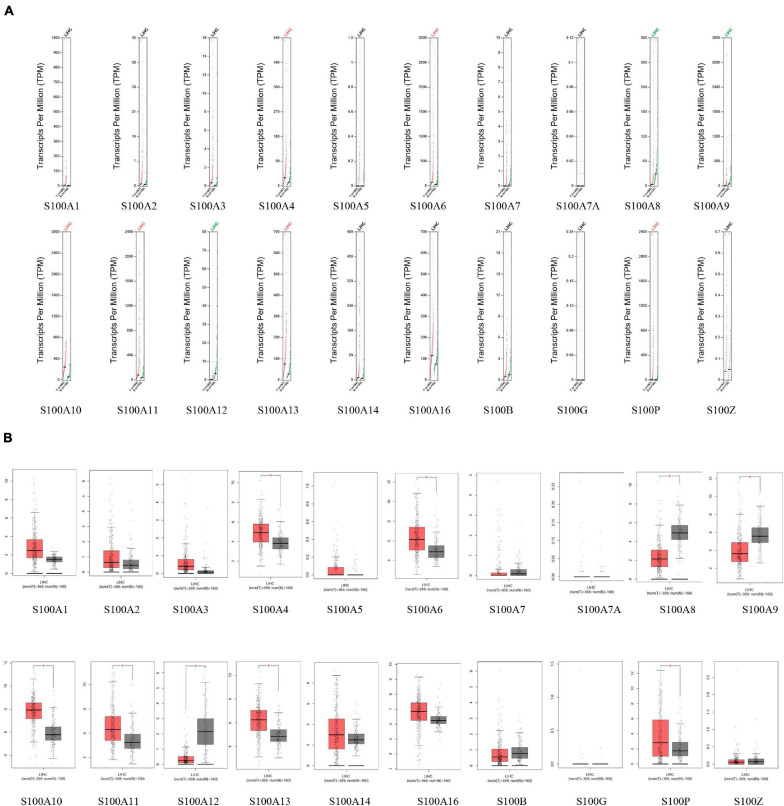
The mRNA levels of S100s in hepatocellular carcinoma (HCC) (GEPIA database). The levels of S100A4/S100A6/S100A10/S100A11/S100A13/S100P were higher in tumor than in normal liver samples, while S100A8/S100A9/S100A12 were downregulated (**A**, scatter diagram; **B**, box plot; *p < 0.01).

**FIGURE 3:**
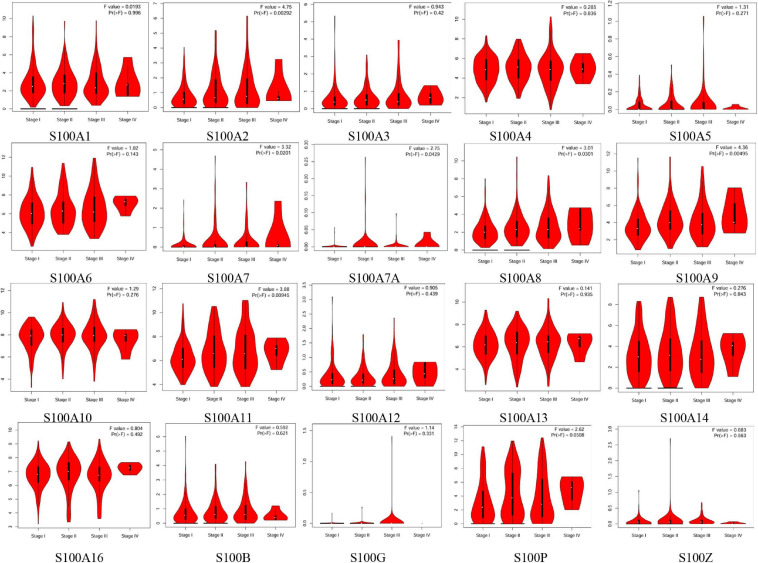
Correlation between S100s mRNA levels and tumor stage in HCC patients (GEPIA database). The mRNA levels of S100A2/S100A7/S100A7A/S100A8/S100A9/S100A11 were correlated with advanced tumor stage. A *p* < 0.05 was considered statistically significant.

**FIGURE 4:**
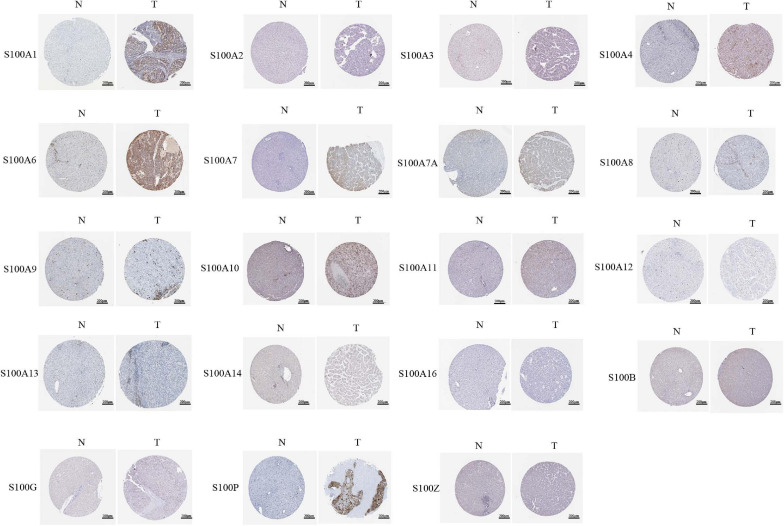
Representative immunohistochemistry images of distinct S100s in HCC tissues and normal liver tissues (Human Protein Atlas). S100A5 protein expression was not shown by the database. Low protein expression of S100A7A was found in normal liver tissues and medium protein expression was observed in HCC tissues. All the other S100s proteins were not detected in normal liver tissues. Low protein expressions of S100A4/S100A11/S100B were observed in HCC tissues. Medium protein expressions of S100A1/S100A6/S100A7/S100A7A/S100A10/S100G/S100P were observed in HCC tissues.

MRNA levels of twenty S100s in cancers were detected by exploring Oncomine databases (Figure 1 and Table 1). The mRNA expression levels of S100A4/S100A6/S100A10/S100A11/S100A13/S100A14/S100P were significantly upregulated in patients with HCC from multiple datasets, while S100A8 were downregulated (*p* = 0.001, fold change = 1.5). However, the other members did not differ significantly in HCC as assessed in the multiple datasets (Chen et al., 2002; Wurmbach et al., 2007; Mas et al., 2009; Roessler et al., 2010). We next utilized the GEPIA dataset to analyze the expression profile of twenty S100s. The gene expression profile analysis demonstrated that the levels of S100A4/S100A6/S100A10/S100A11/S100A13/S100P were higher in tumor than in normal liver samples. Moreover, the transcription levels of S100A8, S100A9, and S100A12 were decreased (*p* = 0.01, Figure 2). Associations between S100s mRNA levels and tumor stage were also analyzed. S100A2/S100A7/S100A7A/S100A8/S100A9/S100A11 groups were shown to be statistically significant (Figure 3). After checking the mRNA levels of S100s in HCC, the Human Protein Atlas was applied to explore the protein expression of S100s in HCC. As displayed in Figure 4, low protein expression of S100A7A was found in normal liver tissues, all the other S100s proteins were not detected in normal liver tissues. S100A5 protein expression was not shown by the database. Low protein expressions of S100A4/S100A11/S100B were observed in HCC tissues. Medium protein expressions of S100A1/S100A6/S100A7/S100A7A/S100A10/S100G/S100P were observed in HCC tissues. Protein expressions of the other members of S100s were not detected in HCC tissues. However, due to the small HCC sample size of immunohistochemistry results in the Human Protein Atlas, the results obtained should be verified further.

**TABLE 1:** Significant changes of S100s expression in transcription level between HCC and normal liver tissues (ONCOMINE database).

	**Types of liver cancer vs. normal tissue**	**Fold change**	***P*-value**	***t*-test**	**Sources and/or References**
S100A1	Hepatocellular carcinoma	−1.94	**8.52E-11**	−6.787	Chen liver
S100A2	Hepatocellular carcinoma	NA	NA	NA	NA
S100A3	Hepatocellular carcinoma	NA	NA	NA	NA
S100A4	Hepatocellular carcinoma	1.77	**3.44E-06**	4.972	Mas liver
S100A5	Hepatocellular carcinoma	NA	NA	NA	NA
S100A6	Hepatocellular carcinoma	2.823	**9.16E-04**	3.502	Roessler liver
	Hepatocellular carcinoma	3.111	**8.84E-14**	9.68	Mas liver
	Hepatocellular carcinoma	3.311	**2.47E-05**	5.093	Wurmbach liver
S100A7	Hepatocellular carcinoma	NA	NA	NA	NA
S100A7A	Hepatocellular carcinoma	NA	NA	NA	NA
S100A8	Hepatocellular carcinoma	−2.672	**8.22E-13**	−7.602	Chen liver
	Hepatocellular carcinoma	−14.197	**3.38E-05**	−5.235	Wurmbach liver
	Hepatocellular carcinoma	−3.959	**9.31E-08**	−6.089	Mas liver
S100A9	Hepatocellular carcinoma	NA	NA	NA	NA
S100A10	Hepatocellular carcinoma	1.998	**3.63E-12**	7.38	Chen liver
	Hepatocellular carcinoma	2.209	**1.27E-04**	4.079	Roessler liver
	Hepatocellular carcinoma	2.341	**1.60E-39**	14.83	Roessler liver
	Hepatocellular carcinoma	2.201	**3.38E-12**	9.544	Mas liver
	Hepatocellular carcinoma	1.795	**3.00E-03**	2.982	Wurmbach liver
S100A11	Hepatocellular carcinoma	2.067	**6.01E-04**	3.58	Roessler liver
	Hepatocellular carcinoma	2.878	**9.50E-13**	9.396	Mas liver
S100A12	Hepatocellular carcinoma	NA	NA	NA	NA
S100A13	Hepatocellular carcinoma	−2.045	**1.58E-04**	-9.281	Chen liver
	Hepatocellular carcinoma	1.506	**5.00E-03**	2.706	Roessler liver
	Hepatocellular carcinoma	1.654	**3.38E-07**	5.733	Mas liver
S100A14	Hepatocellular carcinoma	1.926	**2.46E-13**	9.278	Mas liver
	Hepatocellular carcinoma	2.285	**2.08E-05**	5.326	Wurmbach liver
S100A16	Hepatocellular carcinoma	NA	NA	NA	NA
S100B	Hepatocellular carcinoma	NA	NA	NA	NA
S100G	Hepatocellular carcinoma	NA	NA	NA	NA
S100P	Hepatocellular carcinoma	3.44	**1.26E-09**	6.353	Chen liver
S100Z	Hepatocellular carcinoma	NA	NA	NA	NA

*NA, not available. A *P* value < 0.05 was considered to be statistically significant and was shown in bold print.*

### Prognostic Roles of S100s in HCC

**FIGURE 5:**
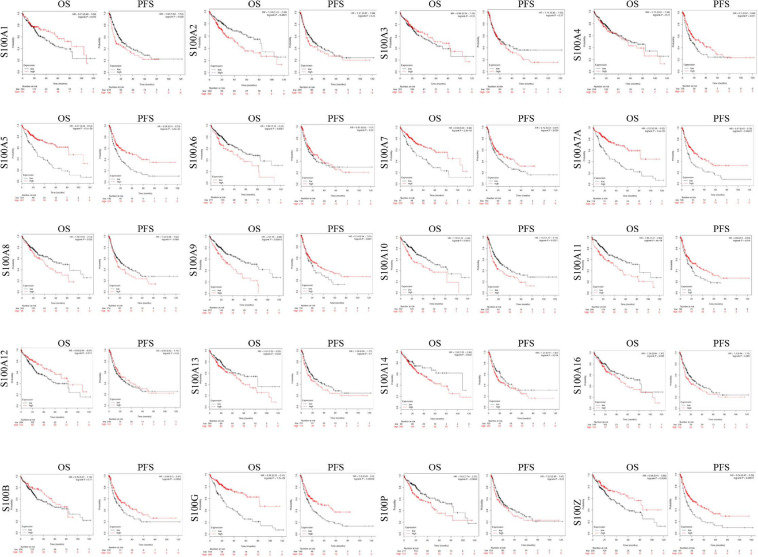
The prognostic value of S100s mRNA expression in HCC patients (Kaplan-Meier Plotter). Higher mRNA expressions of S100A6/S100A10/S100A11/S100A13/S100A14/S100P were shown to have shorter survival time, while higher expression of S100A12 was associated with longer survival time. OS, overall survival; DFS, disease-free survival. A *p* < 0.05 was considered statistically significant.

We explore the prognostic roles of distinct S100s in patients of HCC by using the Kaplan-Meier Plotter tools^8^. As shown in Figure 5 and Table 2, we found that S100A2/S100A6/S100A8/S100A9/S100A10/S100A11/S100A13/S 100A14/S100P high mRNA expressions were significantly associated with poor OS (overall survival). Besides, the high mRNA expressions of S100A5/S100A7/S100A7A/S100A12/S100G/S100Z were correlated with better OS. In addition, the other members of the S100s were found to have no correlations with the patient’s survival. Based on the S100s mRNA expressions profiles mining above, we concluded that higher mRNA expressions of S100A6/S100A10/S100A11/S100A13/S100A14/S100P were found to be significantly associated with shorter OS, while higher expression of S100A12 was associated with favorable OS.

**TABLE 2:** Prognosis roles of S100 family members (analyzed by Kaplan-Meier Plotter tools).

**S100 family members**	**OS**			**PFS**		
	**HR**	**95%CI**	***P***	**HR**	**95%CI**	***P***
S100A1	0.71	0.49–1.04	0.075	1.42	1.04–1.94	**0.028**
S100A2	1.74	1.22–2.49	**0.0021**	1.21	0.87–1.68	0.25
S100A3	0.84	0.59–1.19	0.33	1.14	0.85–1.53	0.37
S100A4	1.17	0.81–1.69	0.41	0.7	0.52–0.94	**0.017**
S100A5	0.37	0.26–0.53	**6.10E-09**	0.54	0.41–0.73	**3.30E-05**
S100A6	1.69	1.16–2.44	**0.0051**	0.87	0.65–1.17	0.35
S100A7	0.48	0.34–0.68	**2.60E-05**	0.72	0.54–0.97	**0.029**
S100A7A	0.37	0.26–0.52	**4.50E-09**	0.57	0.42–0.78	**0.00027**
S100A8	1.48	1.03–2.13	**0.032**	1.32	0.96–1.82	0.088
S100A9	2	1.39–2.88	**0.00013**	0.74	0.54–1.01	0.057
S100A10	1.79	1.25–2.56	**0.0012**	1.57	1.17–2.11	**0.0027**
S100A11	1.85	1.31–2.62	**4.00E-04**	0.68	0.5–0.93	**0.014**
S100A12	0.64	0.44–0.91	**0.014**	0.85	0.62–1.17	0.33
S100A13	1.45	1.03–2.05	**0.034**	1.29	0.95–1.77	0.1
S100A14	1.58	1.01–246	0.42	1.33	0.97–1.83	0.078
S100A16	1.39	0.99–1.97	0.058	1.3	0.96–1.75	0.089
S100B	0.76	0.51–1.13	0.17	0.68	0.5–0.91	**0.0093**
S100G	0.36	0.25–0.51	**1.70E-09**	0.6	0.45–0.8	**0.00058**
S100P	1.63	1.16–2.31	**0.0049**	1.22	0.89–1.67	0.22
S100Z	0.59	0.41–0.86	**0.0049**	0.56	0.41–0.76	**0.00017**

*OS, overall survival; PFS, progression free survival. A *P* value < 0.05 was considered to be statistically significant and was shown in bold print.*

### Genetic Mutations in S100s and Their Associations With Survival of HCC Patients

**FIGURE 6:**
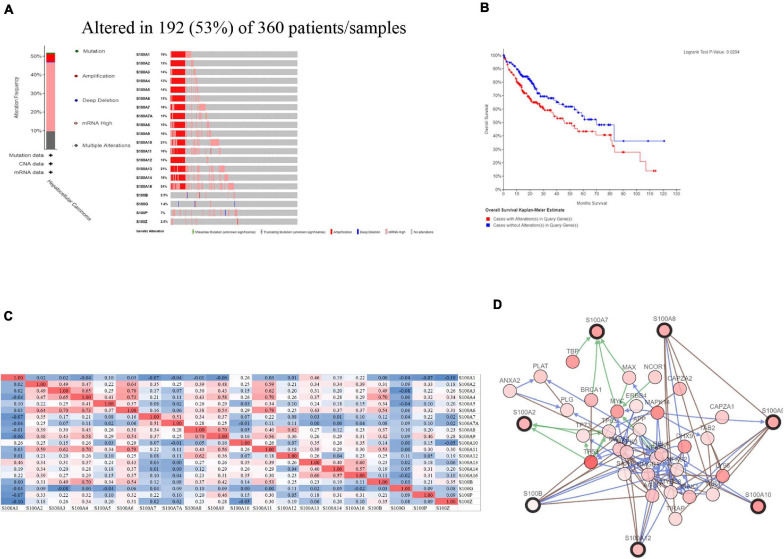
S100s gene expression and mutation analysis in HCC (cBioPortal) **(A)** S100s gene expression and mutation analysis in HCC. **(B)** High genetic alterations (53%) in S100s were associated with shorter OS. **(C)** Correlation between different S100s in HCC (Positive correlations were shown as red color while negative correlations showed blue. The numbers in the cells represented their correlation coefficient). **(D)** The network for S100s and the 50 most frequently altered neighbor genes.

We further explored genetic alteration in S100s and their associations with survival by using the cBioPortal online tool^9^. As shown in Figure 6A, the mutation rate of S100s was really high in HCC patients. In the 360 enrolled samples, genetic mutations were discovered in 192 HCC patients which indicated a 53% mutation rate. S100A16, S100A13, S100A10 were the top three members with genetic mutations (the mutation rates were 24, 21, and 21%, respectively). Furthermore, high rate of genetic alteration in S100s correlated with shorter OS of HCC patients (Figure 6B, *p* = 0.0204). Collectively, the above findings suggested that genetic alteration of S100s may have an impact on prognosis of HCC patients.

### Predicted Functions and Pathways of the Mutations in S100s and Their 50 Frequently Altered Neighbor Genes in HCC Patients

**FIGURE 7:**
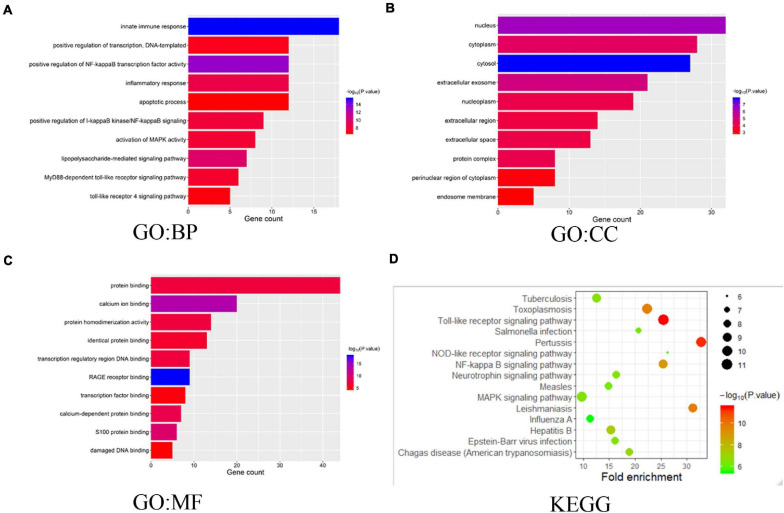
The functions of S100s and genes significantly associated with S100s alterations. GO functional enrichment analysis predicted three main functions of S100s mutations and their 50 frequently altered neighbor genes, including biological process, cellular components and molecular functions **(A–C)**. KEGG pathway analysis on S100s and their 50 most frequently altered neighbor genes was shown at **(D)**.

**FIGURE 8:**
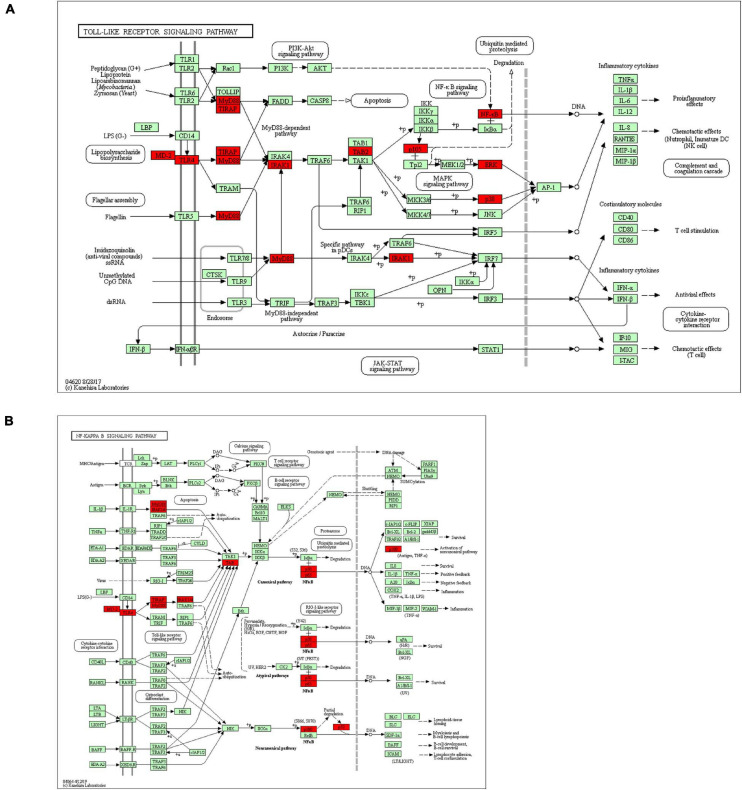
Toll-like receptor signaling pathway and NF-kappa B signaling pathway regulated by the S100s alteration in HCC.

We also explored the S100s correlations and networks with the online tool^10^. As was shown in Figure 6C, significant and positive correlations were observed between differentS100s. Positive correlations were shown as red color while negative correlations showed blue. The numbers in the cells represent their correlation coefficient. We next explored the network for S100s and their 50 most associated genes. As shown in Figure 6D, the apoptotic-related genes, including TP53, TP63,TP73, BRCA1, NFKB1, MAPK1, MAPK3, and MAPK14 were correlated with S100s alterations. Moreover, functions and pathways of these genes were further explored by GO and KEGG in DAVID^11^. As was shown in Figure 7A, biological processes, such as innate immune response, positive regulation of transcription, positive regulation of NF-kappaB transcription factor activity, inflammatory response and apoptotic process were remarkably regulated by the S100s mutations in HCC. As was shown in Figure 7B, cellular components, including nucleus, cytoplasm, cytosol, extracellular exosome, and nucleoplasm were regulated by the S100s mutations in HCC. In addition, S100s mutations also regulated the molecular functions, such as protein binding, calcium ion binding, protein homodimerization activity, identical protein binding and transcription regulatory region DNA binding (Figure 7C). By KEGG analysis, pathways including Toll-like receptor signaling pathway and NF-kappa B signaling pathway (Figures 7D, 8) were found to be associated with S100s alterations.

### The Association of S100s and Immune Infiltration Levels

**FIGURE 9:**
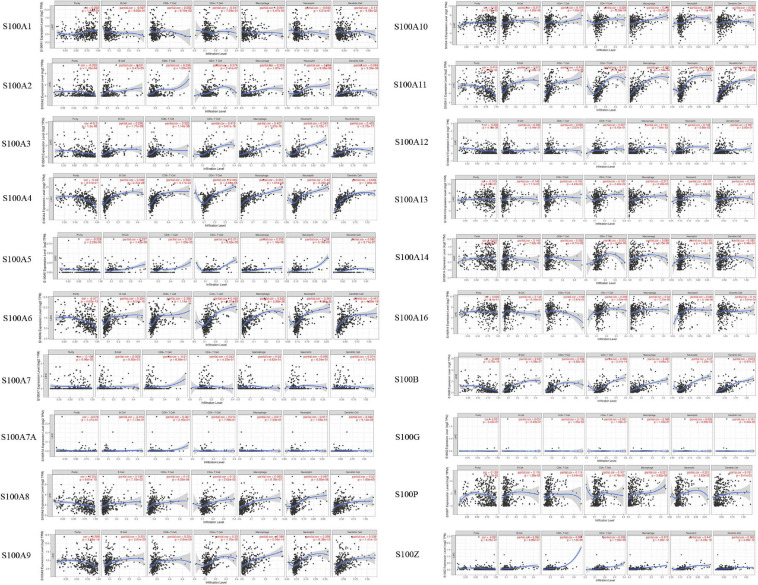
Association of 20 S100s genes with immune infiltration in HCC (TIMER). The expressions of S100A2/S100A3/S100A4/S100A5/S100A6/S100A8/S100A9/S100A10/S100A11/S100A13/S100A14/S100A16/S100B/S100P/S100Z were found to be significantly associated with the immune infiltrating levels of B cells, CD8 + T cells, CD4 + T cells, macrophages, neutrophils, and dendritic cells. A *p* < 0.05 was considered statistically significant.

We further explored the associations between S100s and immune infiltration levels in HCC by applying the TIMER online tool^12^. Spearman tests were used for analyzing the correlation coefficients. As shown in Figure 9, the expressions of S100A2/S100A3/S100A4/S100A5/S100A6/S100A8/S100A9/S100 A10/S100A11/S100A13/S100A14/S100A16/S100B/S100P/S100Z were all shown to be closely associated with all six immune infiltrates (B cells, CD8 + T cells, CD4 + T cells, macrophages, neutrophils, and dendritic cells). For instance, S100A4 expression had significant positive correlations with all six immune infiltrates, especially B cells (*r* = 0.548, *P* = 2.37E-28), CD8 + T cells (*r* = 0.564, *P* = 4.17E-30), macrophages (*r* = 0.551, *P* = 1.61E-28), and dendritic cells (*r* = 0.665, *P* = 7.85E-45). S100A6 expression had significant positive correlations with all six immune infiltrates, especially macrophages (*r* = 0.532, *P* = 2.88E-26). S100A11 expression had significant positive correlations with all six immune infiltrates, especially dendritic cells (*r* = 0.566, *P* = 3.18E-30). S100B expression had significant positive correlations with all six immune infiltrates, especially B cells (*r* = 0.537, *P* = 4.56E-27), CD8 + T cells (*r* = 0.528, *P* = 6.62E-26) and dendritic cells (*r* = 0.616, *P* = 6.97E-37). Next, the associations between S100s and gene markers of different immune infiltrates were also investigated. As shown in Supplementary Table 1, significant associations between the S100s and markers of immune cells were found. In short, all the results above suggested that S100s played a critical role in immune infiltration of HCC.

## Discussion

S100s dysregulation has been seen in a variety of human malignancies (Allgower et al., 2020). Although the role of some members of S100s in the tumorigenesis and prognosis of several cancers has been partially confirmed, comprehensive understanding the role of twenty S100s in HCC has yet to be conducted. Our work aims to explore the expression, mutation, prognosis and associations with immune infiltration of S100s in HCC. We expect that our results will be helpful in improving existing knowledge, advancing treatment designs, and prognosis prediction.

In this study, our results showed that the mRNA levels of S100A4/S100A6/S100A10/S100A11/S100A13/S100A14/S100P were significantly upregulated in HCC patients, while the S100A8/S100A9/S100A12 mRNA levels were decreased. Survival analysis indicated that higher mRNA expressions of S100A6/S100A10/S100A11/S100A13/S100A14/S100P were shown to have shorter OS, while higher expression of S100A12 was associated with longer OS. Moreover, high mutation rate (53%) was found in S100s and correlated with poor survival. These findings suggested that S100A6/S100A10/S100A11/S100A12/S100A13/S100A14/S100P may be used as new prognostic or therapeutic biomarkers.

S100A4 is a well-known tumor promoting gene and is the most studied one in HCC. Overexpression of S100A4 led to heavier tumors and more metastasis sites, while down-regulation of S100A4 resulted in a reduction of proliferation and invasion of HCC cells (Yan et al., 2013). S100A4 regulates invasion and migration of HCC cells via NF-kappaB-dependent MMP9 signal (Zhang et al., 2013). It was also reported that S100A4 levels were significantly increased in HCC samples compared with controls, moreover, S100A4 protein levels correlated with tumor differentiation, invasion, recurrence and overall survival (Liu et al., 2013). Interestingly, in our study, we showed that both mRNA and protein expression of S100A4 were significantly higher in tumor tissues compared with non-tumor tissues. However, the S100A4 mRNA expression had no correlation with survival time in patients with HCC. One possible reason maybe the protein levels of S100A4 were elaborately controlled by post-transcriptional regulation. And the conclusion obtained here should be verified in further studies.

S100A6 is another well-studied member of S100s in cancer. S100A6 is upregulated in many kinds of tumors and may take part in regulating cell proliferation, apoptosis, cytoskeleton dynamics and migration (Bresnick et al., 2015). It was reported that S100A6 was overexpressed in HCC tissues compared with non-tumor tissues and the expression of S100A6 correlated with poor differentiation (Hua et al., 2011). S100A6 enhanced the proliferation and migration of HCC cells via the activation of PI3K/AKT pathway (Li et al., 2014). In our study, the results showed that the expression of S100A6 was significantly upregulated in HCC tissues than in para-tumor tissues. Moreover, high S100A6 mRNA expression correlated with poor prognosis.

S100A10 is an oncogenic event in various malignancies, including thyroid anaplastic carcinoma, colorectal cancer and ovarian cancer (Ito et al., 2007; Suzuki and Tanigawara, 2014; Lokman et al., 2016). The role of S100A10 in HCC has rarely been explored. DNA microarray analysis of HCC identified S100A10 as one of the key members linked to integrin and Akt/NF-kappaB signaling (Kittaka et al., 2008). Shan et al. (2013) reported that miR-590-5p inhibited proliferation of HCC cells via down-regulating S100A10 expression. The expression of S100A10 in HCC cells was significantly increased than normal hepatic cells (Zhao et al., 2020). In our study, the results showed that the expression of S100A10 was significantly upregulated in HCC tissues than in para-tumor tissues. Moreover, high S100A10 mRNA expression correlated with poor prognosis.

S100A11 has dual roles in cell growth regulation, intracellular S100A11 inhibits cell growth and extracellular S100A11 promotes cell proliferation (Sakaguchi and Huh, 2011). S100A11 is reported to be associated with advanced tumor behavior and poor survival in bladder cancer (Yao et al., 2007). S100A11 activates Wnt/beta-catenin Signaling and promotes proliferation and migration of cervical cancer cells (Meng et al., 2019). In HCC, S100A11 was found to be a metastasis-associated protein by proteomic analysis (Song et al., 2006). Overexpression of S100A11 promotes migration of HCC cells (Luo et al., 2013). A recent study showed that S100A11 overexpression promoted inflammation/fibrosis and was associated with tumor grade and poor survival time of HCC patients (Sobolewski et al., 2020). In our study, the results showed that the expression of S100A11 was significantly upregulated in HCC tissues than in para-tumor tissues. Moreover, high S100A11 mRNA expression correlated with advanced tumor stage and poor prognosis.

S100A12 is commonly expressed on neutrophils monocytes and macrophages, the plasma levels of S100A12 are systemic biomarkers for many inflammatory diseases (Meijer et al., 2012). S100A12 is dysregulated in many types of cancers, upregulated in colorectal cancer and papillary thyroid cancer but reduced in gastric carcinoma and oropharyngeal squamous cells (Thierolf et al., 2008; Funk et al., 2015; Li et al., 2016; Wang et al., 2020). Studies on S100A12 in HCC are limited. Proteomics analysis indicated that S100A12 might be useful marker for predicting the early recurrence/metastasis of HCC after resection (Huang et al., 2014). Another study revealed that S100A12 was expressed not on HCC cells but intratumoral stroma cells and S100A12 overexpression predicted poor prognosis (Cai et al., 2018). In this study, the results showed that the mRNA expression of S100A12 in HCC tissues was significantly lower than that in normal tissues. Moreover, high S100A12 expression correlated with favorable OS in patients with HCC.

There are limited studies on S100A13 in cancer. S100A13 was first identified as a powerfully angiogenic biomarker in some kinds of tumor such as melanoma and gliomas (Landriscina et al., 2006; Massi et al., 2010). A recent study showed that S100A13 overexpression promoted migration of lung cancer cells (Pierce et al., 2008). Consistent with these results, the results showed that the expression of S100A13 was significantly upregulated in HCC tissues than in para-tumor tissues. Moreover, high S100A13 mRNA expression correlated with poor prognosis.

S100A14, a novel member of the S100s, has been characterized by differentially expressed and functioned in various cancer types. Overexpression of S100A14 has been found in some kinds of cancers, such as breast cancer, lung cancer and bladder cancer. But downregulated in some other kinds of cancers, for example colorectal carcinoma and renal cancer (Basnet et al., 2019). S100A14 activates ERK and NF-κB signaling and promotes ESCC cell proliferation. However, in oral squamous cell carcinoma, S100A14 positively affects the function of p53 and inhibits cell proliferation (Jin et al., 2011; Sapkota et al., 2012). Few studies investigated the role of S100A14 in HCC. Zhao et al. (2013) reported that knock-down of S100A14 decreased proliferation and invasion of HCC cells. Similar to these results, the results showed that the expression of S100A14 was significantly upregulated in HCC tissues than in para-tumor tissues. Moreover, high S100A14 mRNA expression correlated with poor prognosis.

S100P is an oncogenic event in many types of tumors, including pancreatic adenocarcinoma, breast cancer and ovarian cancer (Wang et al., 2006; Ezzat et al., 2016; Bai et al., 2018). In ovarian cancer, S100P was shown to be positively correlated with CA125 and poor prognosis (Wang et al., 2015). Studies investigating the role of S100P in HCC were limited. S100P was found to be overexpressed in HCC tissues and correlated with advanced tumor behavior and poor prognosis (Yuan et al., 2013). Another study showed that S100P was useful predictor for microvascular invasion and portal vein tumor thrombus (Qi et al., 2021). Similar to these results, our findings showed that the expression of S100P was significantly upregulated in HCC tissues than in para-tumor tissues. Moreover, high S100P mRNA expression correlated with poor prognosis.

HCC is now generally considered as an immunogenic tumor (Pardee and Butterfield, 2012). Various immune cells have been proved to affect the progression and prognosis of HCC. For instance, tumor-associated macrophages (TAMs) and tumor infiltrations of neutrophils are associated with poor prognosis in patients with HCC, while CD8 + cytotoxic T lymphocytes (CTLs) and dendritic cells (DCs) were predictive for better prognosis (Chen et al., 2020). In this study, our results indicated that S100s played a critical role in immune infiltration of HCC. Especially, both S100A4 and S100A6 had strong positive correlations with macrophages. Therefore, the combination these S100s inhibitors with immunotherapies may be a promising strategy in HCC therapy.

Nevertheless, our study had some limitations. First, all the data included for analysis were retrieved from online databases, further researches are still required to verify these findings. Second, all survival analyses were conducted by the Kaplan-Meier database, the independent prognostic values of S100s by multivariate analysis were not acquired because of data limitation. Third, exploring the detailed mechanism of each S100s in HCC was not conducted in this research, future researches are still needed.

In conclusion, in this study, we systemically explored the expression, mutations, predictive signaling pathways, prognosis and associations with immune infiltration of different S100s in patients with HCC. Our research gives new insights regarding the roles of S100s to HCC and may be helpful for the further combination of immunotherapies with S100s inhibitors in HCC.

## Data Availability Statement

The datasets presented in this study can be found in online repositories. The names of the repository/repositories and accession number(s) can be found in the article/Supplementary Material.

## Ethics Statement

Written informed consent was obtained from the individual(s) for the publication of any potentially identifiable images or data included in this article.

## Author Contributions

BZ and XXie contributed to the study design and critical revision of the manuscript. SZ, LL, and TX carried out the study and drafted the manuscript. CJ, XXu, YW, and MW analyzed the data. All authors read and approved the final manuscript.

## Conflict of Interest

The authors declare that the research was conducted in the absence of any commercial or financial relationships that could be construed as a potential conflict of interest.

## Footnotes

1 https://www.oncomine.org

2 http://gepia.cancer-pku.cn/

3 https://www.proteinatlas.org

4 www.kmplot.com

5 www.cbioportal.org

6 https://david.ncifcrf.gov/

7 https://cistrome.shinyapps.io/timer/

8 http://kmplot.com/analysis/

9 www.cbioportal.org

10 www.cbioportal.org

11 https://david.ncifcrf.gov/summary.jsp

12 https://cistrome.shinyapps.io/timer/

## Supplementary Material

Supplementary Table 1 Correlations between S100s genes and markers of immune cells in HCC.
